# Salidroside protects against ox-LDL-induced endothelial injury by enhancing autophagy mediated by SIRT1-FoxO1 pathway

**DOI:** 10.1186/s12906-019-2526-4

**Published:** 2019-05-30

**Authors:** Zhongsheng Zhu, Jinyu Li, Xiaorong Zhang

**Affiliations:** Department of Cardiology, Shanghai Pudong Hospital, Fudan University Pudong Medical Center, No.2800 Gongwei Road, Pudong New District, Shanghai, 201399 China

**Keywords:** Atherosclerosis, Salidroside (SAL), Oxidized low-density lipoprotein (ox-LDL), Endothelial cell, Oxidative stress, Autophagy

## Abstract

**Background:**

Atherosclerosis is a condition with the vascular accumulation of lipid plaques, and its main major contributing factor is endothelial injury induced by oxidized low-density lipoprotein (ox-LDL). Salidroside (SAL) is the primary active ingredient of *Rhodiola rosea*, and exhibits antioxidant properties on endothelial cells and alleviates atherosclerosis. However, the effect of SAL on autophagy in ox-LDL-induced vascular endothelial injury remains unclear. Here, we investigated the effect and underlying mechanisms of SAL on autophagy in human umbilical vein endothelial cells (HUVECs).

**Methods:**

HUVECs were incubated with ox-LDL to induce in vitro atherosclerosis model. The cell viability and injury were evaluated by cell counting kit-8 (CCK-8) assay and lactate dehydrogenase (LDH) release assay. The oxidative stress was evaluated by NADPH oxidase, malondialdehyde (MDA) and superoxide dismutase (SOD) activities. Immunofluorescence was performed to detect autophagy using LC3β antibody. Quantitative real-time PCR (qRT-PCR) and western blot were performed to measure the mRNA expressions of SIRT1 and Forkhead box O1 (FOXO1). Nicotinamide (NAM) and AS1842856 were used to inhibit activities of SIRT1 and FOXO1, respectively.

**Results:**

Exposure of HUVECs to ox-LDL (100 μg/mL) reduced cell viability, increased cellular MDA, and reduced SOD in a concentration-dependent manner. The pretreatment with SAL (20, 50 and 100 μM) significantly enhanced the cell viability and decreased LDH release in HUVECs exposed to ox-LDL (100 μg/mL). ox-LDL induced autophagy in HUVECs, which was further enhanced by pretreatment with SAL. However, SAL attenuated increase in oxidative stress in HUVECs induced by ox-LDL. ox-LDL reduced mRNA and protein expressions of SIRT1 and FOXO1, which could be reversed by SAL. The protective, anti-oxidative and pro-autophagic effects of SAL could be obviously abolished by cotreatment with SIRT1 inhibitor or FOXO1 inhibitor.

**Conclusion:**

Salidroside shows protective effect on endothelial cell induced by ox-LDL, and the mechanisms might be related to autophagy induction via increasing SIRT1 and FoxO1 expressions.

## Background

Atherosclerosis is characterized by accumulation of lipid plaques in vascular endothelium [1]. Endothelial injury is initial event and contributing factor of atherosclerosis, and is mainly caused by oxidized low-density lipoprotein (ox-LDL) [2]. ox-LDL destroys the oxidation-reduction equilibrium of vascular endothelial cells and induces apoptosis of endothelial cells, thus contributing to endothelial injury [3]. Oxidative stress promotes low density lipoprotein (LDL) oxidation of the vascular wall by increasing the superoxide anion, and large amount of ox-LDL further damages vascular endothelium [4]. Endothelial injury is the initial but reversible step in the development of atherosclerosis [5]. Therefore, prevention of endothelial injury has become an promising therapeutic strategy for reversing atherosclerosis.

Autophagy is a highly regulated metabolic process in which long lived proteins and organelles are degraded through the lysosomal system in unfavorable environment. Autophagy is involved in variety of physiological and pathological conditions, including oxidative stress, inflammation, starvation and immune responses [6]. Autophagy plays essential roles in homeostasis and function of heart and vessel, and defective or excessive autophagy leads to atherosclerosis and other cardiovascular disorders [7]. In fact, autophagy shows both protective and aggravating effects on vascular injury in atherosclerosis. Autophagy participates in the defense mechanism against oxidative stress, thereby preventing vascular cell apoptosis [8]. Autophagy also destroys most cytosols and organelles, ultimately leading to endothelial cell death (autophagy death) and plaque instability [9]. Therefore, the precise role of autophagy in the treatment of atherosclerosis by various agents should be investigated in different in vitro systems and animal models.

SIRT1 is a member of the NAD + -dependent deacetylases, and SIRT1 deficiency in endothelial cells promotes oxidative stress, inflammation, foam cell formation, and increased progression of atherosclerosis [10]. SIRT1 is also a promotor of autophagy and SIRT1 inhibition accelerate atherosclerotic plaque development through impaired autophagy in ApoE (−/−) mice [11]. Forkhead box O1 (FOXO1) is a transcription factor and involves a series of intracellular functions, including autophagy, mitochondrial dysfunction and apoptosis [12]. FOXO1 is a potent inhibitor of oxidative stress and thus considered as a therapeutic target for diseases with excessive oxidative stress [13]. FOXO1 is strongly expressed in atherosclerotic plaques and shows atheroprotective effect, as FOXO1 silencing in endothelial cells prevented atherosclerosis in mouse model [14]. Until now, the roles of SIRT1 and FOXO1 in atherosclerosis as therapeutic target remain largely unknown.

Salidroside (SAL) is the main ingredient of *Rhodiola rosea*, with suppressive effects on oxidative stress [15]. Salidroside protected foam cells against injury induced by ox-LDL, and alleviated atherosclerosis in apoE(−/−) mice [16, 17]. Salidroside induced autophagy and decreased apoptosis in HUVECs exposed to ox-LDL [18]. However, whether SIRT1 and FOXO1 mediate autophagy by salidroside remains unclear.

In this study, we investigated the underlying mechanism of the protective effects and autophagy of salidroside on ox-LDL-induced endothelial cell injury. We hypothesized that activation of SIRT1-FoxO1 pathway mediates autophagy induction, and reduced oxidative stress and endothelial cell injury by salidroside.

## Methods

### Reagents and chemicals

Human umbilical vein endothelial cells (HUVECs) were purchased from Cell Bank of Chinese Academy of Sciences (Shanghai, China). Salidroside (purity > 99%, CAS: 43866), 3-Methyladenine (3-MA, CAS: M9281) and nicotinamide (NAM, CAS: 72340) were purchased from Sigma-Aldrich (Merck KGaA, Darmstadt, Germany). FOXO1 inhibitor AS1842856 (CAS: A15871) was purchased from AdooQ BioScience (Irvine, CA, USA). Ox-LDL was purchased from Beijing Solarbio Life Science Company (No. H7950; Beijing, China). Low glucose DMEM media and fetal bovine serum (FBS) were purchased from Invitrogen (Carlsbad, CA, USA). Cell counting kit-8 (CCK-8) kits was obtained from Beyotime Institute of Biotechnology (No. C0038; Shanghai, China). LDH assay kit (CAS: A020–3), MDA assay kit (CAS: A003–1), SOD assay kit (CAS: A001–1) and NADPH oxidase kit (CAS: A127) were purchased from Nanjing Jiancheng Bioengineering Institute (Nanjing, China). The primary antibodies against LC3β (CAS: sc-398,822), SIRT1 (CAS: sc-74,504) and FOXO1 (CAS: sc-374,427) were purchased from Santa Cruz Biotechnology (Santa Cruz, CA, USA).

### Cell culture and treatment

HUVECs were cultured in DMEM (low glucose) supplemented with 10% FBS, and maintained in a humidified atmosphere containing 5% CO_2_ at 37 °C. In vitro atherosclerosis model was established by incubation of HUVECs with different concentrations of ox-LDL (0, 10, 20, 50, 100, 150 μg/mL) for 48 h. Then 100 μg/mL of ox-LDL was chosen as the propriate concentration and HUVECs were pretreated with salidroside at 20, 50, 100 μM for 2 h and then exposed to ox-LDL (100 μg/mL) for 48 h. The control group received 0.1% DMSO as vehicle.

### Cell viability assay

Cell viability was measured by cell counting kit-8 (CCK-8). HUVECs were seeded in 96-well plates at a density of 1 × 10^4^ cells/mL, and then treated with different concentrations of ox-LDL (0, 10, 20, 50, 100, 150 μg/mL), ox-LDL (100 μg/mL) and SAL (20, 50, 100 μM) for 48 h. After washing three times with PBS, cell were incubated with CCK-8 solution and media (1:10 dilution) at 37 °C for 1 h, and then measured absorbance at 450 nm by a microplate reader.

### LDH release assay

HUVECs were cultured in 96-well plates at a density of 1 × 10^4^ cells/mL. HUVECs were pretreated with salidroside at 20, 50, 100 μM for 2 h and then exposed to ox-LDL (100 μg/mL) for 48 h, then the LDH content in the media was assessed using a LDH activity kit.

### Determination of NADPH oxidase, MDA and SOD activities

HUVECs were plated (1 × 10^5^/mL) in 6-well plates with DMEM medium containing 1% FBS. After 24 h, HUVECs received different concentrations of ox-LDL, or ox-LDL (100 μg/mL) and salidroside (20, 50, 100 μM), or ox-LDL (100 μg/mL), salidroside (100 μM) and 3-MA, NAM or AS1842856, and cells were cultured for additional 48 h. Then cells were harvested, lysed in PBS by ultrasonic pyrolysis and centrifuged at 3000×g for 10 min at 4 °C. A total of 100 μL supernatant and were mixed with detection working fluid for NADPH oxidase, MDA or SOD at 37 °C for 15 min. Then the reaction mixture was centrifuged and transferred to 96-well plates, and a microplate reader was used to measure absorbance values at 340 nm (NADPH oxidase), at 532 nm (MDA), and at 520 nm (SOD).

### Immunofluorescence

HUVECs (2 × 10^4^/mL) were seeded on circular coverslips in 6-well culture plates. In one experiment, cells were pretreated with salidroside at 20, 50, 100 μM for 2 h and exposed to ox-LDL (100 μg/mL) for further 48 h. In another experiment, cells were incubated with salidroside (100 μM) and SIRT1 inhibitor nicotinamide (NAM, 100 nM) or FOXO1 inhibitor (AS1842856, 50 nM) for 2 h, an then incubated with ox-LDL (100 μg/mL) for further 48 h. After washing with PBS for 3 times, cultures were fixed 4% paraformaldehyde (pH 7.4) for 20 min, and blocked with 1% BSA and 0.1% Triton-X-100 for 10 min at room temperature. HUVECs were incubated with goat polyclonal anti-LC3β antibody at 4 °C overnight, followed by PBS washing and incubation with fluorescein isothiocyanate (FITC) conjugated secondary antibody (IgG) for 1 h at 37 °C. After rinsing several times, the cells were incubated with DAPI (10 mg/mL) for 5 min at room temperature. Cultures were then mounted on glass slides and observed under a confocal microscope (Leica, Germany). The number of cells with punctate fluorescent LC3 was counted, and normalized to all DAPI fluorescent cells (For each group a minimum of 100 fluorescence-positive cells were counted), and presented as a percentage of cells with LC3 dots.

### Quantitative real-time PCR (qRT-PCR)

Total RNA was isolated using TRIzol® reagent reversely transcribed into cDNA using Superscript II reverse transcriptase (Toyobo Life Science, Osaka, Japan). The qRT-PCR reaction system contained 2 μL of cDNA sample solution, 10 μL of SYBR-Green PCR master mix, 0.5 μL of forward and reverse primers (1 μM), and 7.5 μL of H_2_O. The primer sequences were as follows: SIRT1, forward 5′-GCC AGA GTC CAA GTT TAG AAG A-3′, reverse 5′-CCA TCA GTC CCA AAT CCA G-3′; FOXO1, forward 5′-GGC TGA GGG TTA GTG AGC AG-3′ and reverse 5′-AAA GGG AGT TGG TGA AAG ACA-3′ and GAPDH, forward 5′-CCT CAA GAT CAT CAG CAA TG-3′ and reverse 5′-CCA TCC ACA GTC TTC TGG GT-3′. The amplification process was carried out as follows: denaturation at 95 °C for 5 min, followed by 40 cycles of denaturation at 95 °C for 45 s, annealing at 50 °C for 45 s and elongation at 72 °C for 45 s, with the final extension step maintaining at 72 °C for 10 min. qRT-PCR was performed using an ABI Prism 7500 Fast Real-time PCR instrument (Applied Biosystems; Foster City, CA, USA), and analyzed using the 2-ΔΔCt method. The mRNA levels of SIRT1 and FOXO1 were normalized to those of GAPDH to assess the significance of the differences between the groups.

### Western blotting

Total protein was extracted from homogenate samples of HUVECs, the lysate was centrifuged to collect supernatant, and the protein concentration was determined by bicinchoninic acid assay. Total protein (50 μg) was loaded in 10% SDS-PAGE and then transferred to a nitrocellulose membrane. The membrane was then blocked with 5% low fat milk and 0.05% Tween-20 in Tris buffered saline. Subsequently, the membrane was incubated with primary antibody (diluted 1:200) as mouse anti-SIRT1 and anti-FOXO1. After complete washing, the membrane was incubated with horseradish peroxidase-conjugated anti-mouse secondary antibody (1:250). The bands identified by the primary antibody were observed by a chemiluminescent detection system (ECL, Amersham Life Sciences, Buckinghamshire, UK). The optical density of the protein bands was analyzed using ImageJ software. The density values of SIRT1 and anti-FOXO1 were normalized to β-actin.

### Statistical analysis

Data are presented as means ± standard deviation (SD), and analyzed by SPSS 19.0 statistical software. Comparisons between three or more groups were analyzed by analysis of variance (ANOVA), followed by the student Newman-Keuls (SNK) test. *P* < 0.05 was considered as statistical significance.

## Results

### Salidroside prevented the cytotoxic activity of ox-LDL in HUVECs

**Fig. 1:**
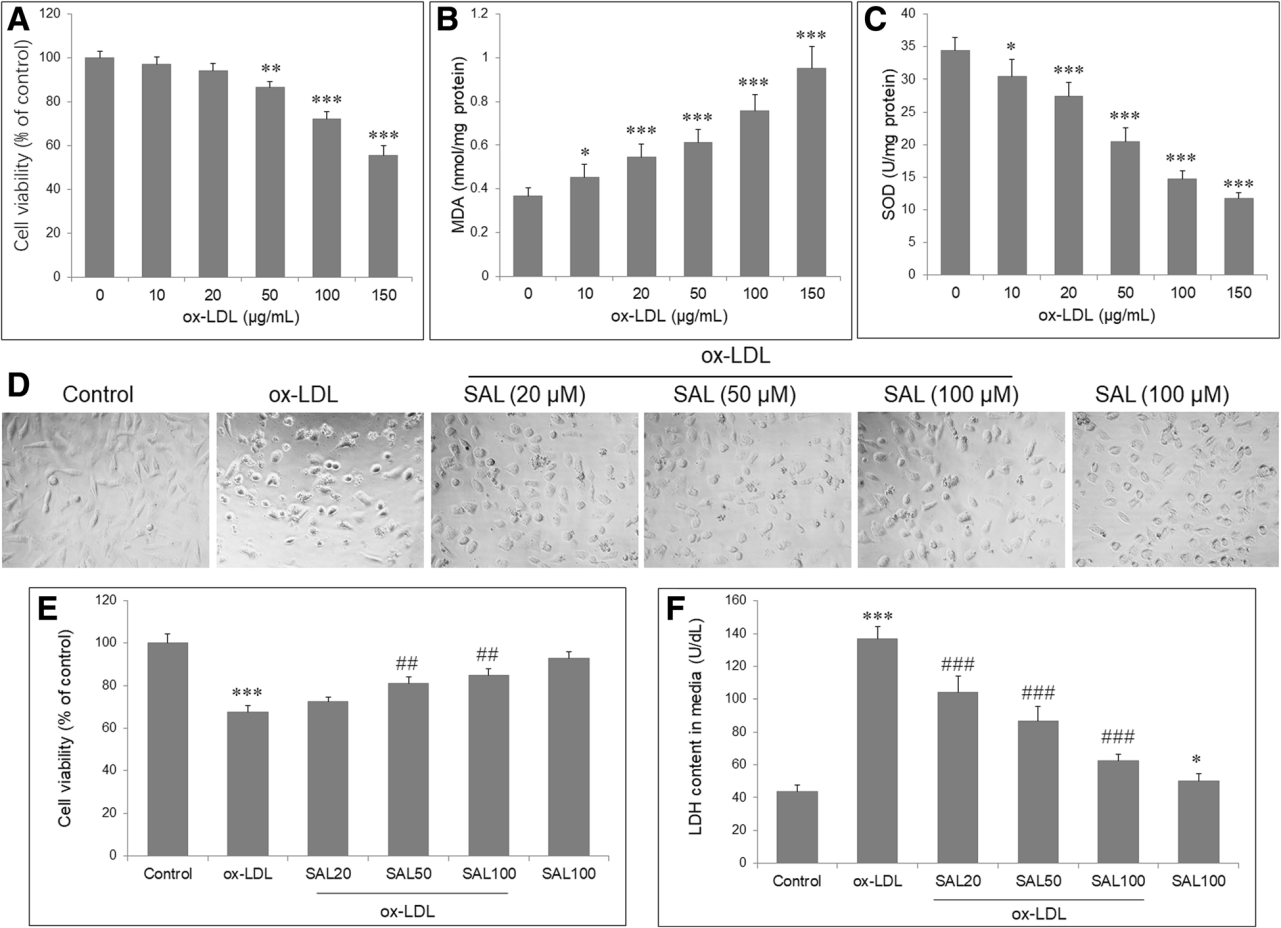
Effect of salidroside on endothelial cell injury. HUVECs were exposed to various concentrations of ox-LDL (10, 20, 50, 100 and 150 μg/mL) for 48 h. **a** Cellular viability was detected by CKK-8 assay, which was normalized to control group. MDA level (**b**) and SOD enzymatic activity (**c**) in HUVECs were analyzed by chromometry using commercially available assay kits. **d** HUVECs were pretreated with SAL (20, 50, 100 μM) for 2 h, and then incubated with ox-LDL (100 μg/mL) for 48 h. HUVEC morphology was observed under an inverted phase contrast microscope (× 10). After 48 h of ox-LDL treatment, HUVECs demonstrate typical cellular fragmentations, vacuoles, and debris, which are obviously attenuated by SAL. SAL significantly attenuated the decrease cellular viability (**e**) and increase in LDH release (**f**) in HUVECs exposed to ox-LDL. Data are shown as mean ± SD (*n* = 6). *P < 0.05, **P < 0.01, ***P < 0.001 vs. control group; ##*P* < 0.01, ###*P* < 0.001 vs. ox-LDL group

In order to establish the in vitro atherosclerosis model, the HUVECs were exposed to various concentrations of ox-LDL (10, 20, 50, 100 and 150 μg/mL) for 48 h. The cultured HUVECs displayed a reduction of cell viability dependent on various concentrations of ox-LDL, with significant reduction in cell viability at 50, 100 and 150 μg/mL (Fig. 1a). ox-LDL also induced oxidative injury in HUVECs, as evidenced by remarkably increased MDA and decreased SOD in all concentrations (Fig. 1b, c). Therefore, exposure to ox-LDL at 100 μg/mL was used in subsequent experiments. We then examined the effect of SAL on endothelial cell, and HUVECs were pretreated with SAL at 20, 50, 100 μM for 2 h and exposed to ox-LDL (100 μg/mL) for further 48 h. HUVEC morphology was observed under an inverted phase contrast microscope (× 10). ox-LDL treatment for 48 h demonstrate cellular fragmentations, vacuoles and debris, and these abnormal cellular morphologies were attenuated by SAL pretreatment, with more obvious improvement by SAL at 100 μM compared with other two concentrations (Fig. 1d). CKK-8 assay showed that pretreatment with SAL significantly increased the cell viability at 20 and 50 μM and suppressed the LDH release at 10, 20 and 50 μM (Fig. 1e, f). However, SAL alone (100 μM) had no significant effect on cell viability, and slightly increased LDH release in HUVECs without ox-LDL.

### Salidroside promoted autophagy and suppressed oxidative stress in HUVECs with ox-LDL

**Fig. 2:**
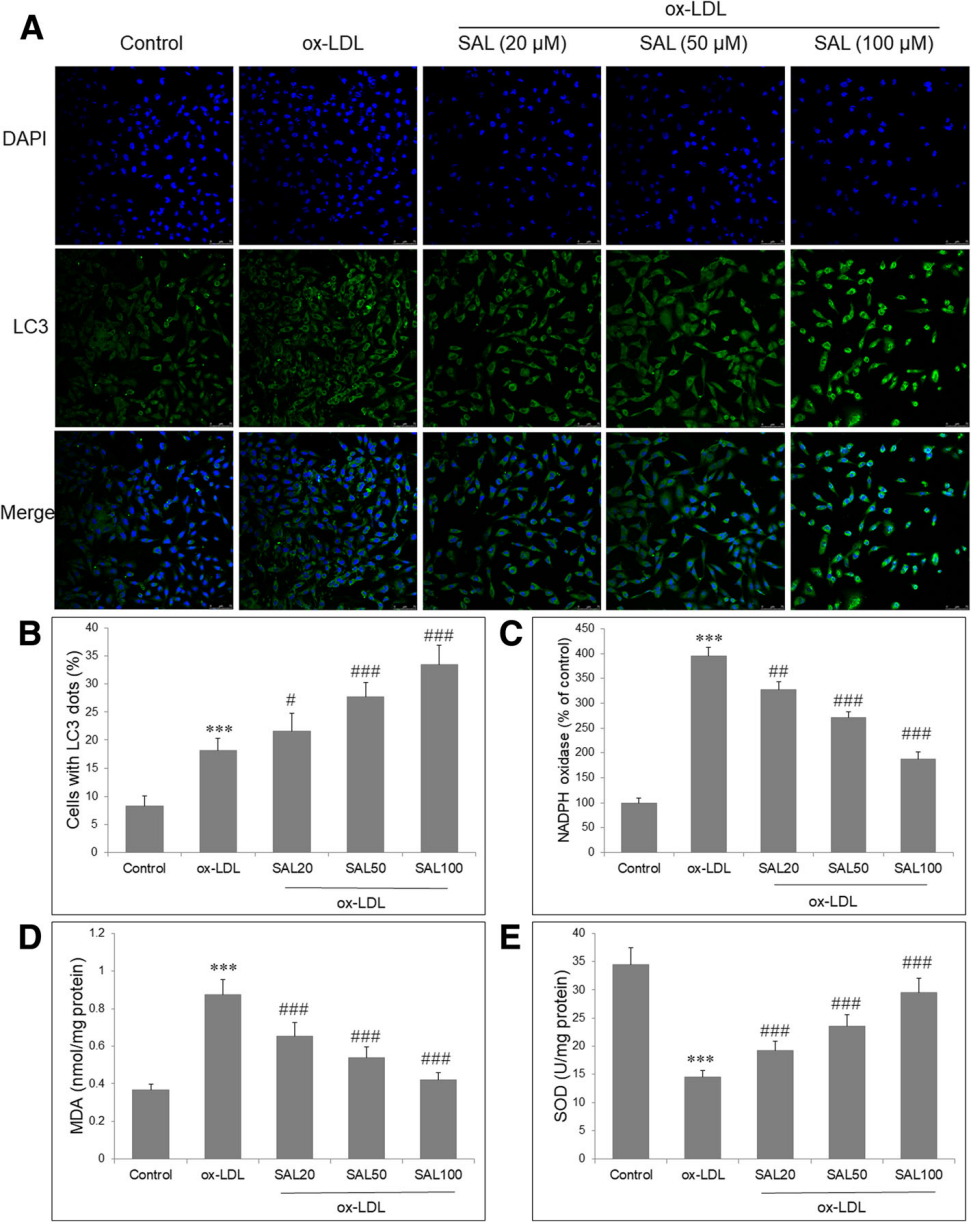
Effect of salidroside on autophagy and oxidative stress in ox-LDL-induced HUVECs. **a** Fluorescence microscopy after immunofluorescence staining with an LC3 antibody, followed by FITC-labeled secondary antibody (green, original magnification × 100). The nucleus was stained with DAPI (blue). **b** Quantification of fluorescence intensity of LC3, as presented by cells with LC3 dots. SAL decreases the NADPH oxidase activity (**c**), increases MDA level (**d**) and decreases SOD activity (**e**) in HUVECs exposed to ox-LDL (100 μg/mL). Data are shown as mean ± SD (*n* = 6). ***P < 0.001 vs. control group; #*P* < 0.05, ##*P* < 0.01, ###*P* < 0.001 vs. ox-LDL group

To assess the regulation of SAL on autophagy in ox-LDL treated HUVECs, we monitored the extent of autophagy by staining HUVECs with LC3 antibody by confocal microscopy. The FITC-positive green puncta mainly show autophagosomes, whereas the DAPI-positive blue puncta represent nucleus. The green puncta was merged with the blue puncta and appeared yellow images, which were indicators of autophagosomes. ox-LDL induced autophagy in HUVECs, as evidenced by slightly increased yellow fluorescence. SAL pretreatment further increased yellow fluorescence (Fig. 2a). Quantification analysis showed that SAL significantly increased the percentage of cells with LC3 dots (Fig. 2b). In order to investigate the effect of SAL on oxidative stress, we measured intracellular NADPH oxidase activity, MDA level and SOD activity. ox-LDL significantly increased NADPH oxidase activity (Fig. 2c) and MDA level (Fig. 2d), and decreased SOD activity (Fig. 2e) in HUVECs. However, these oxidative stress indicators could be significantly reversed by SAL in a concentration-dependent manner in ox-LDL-induced HUVECs (*P* < 0.05). Taken together, ox-LDL induced autophagy and oxidative stress in HUVECs, and SAL could further promote autophagy but exert an antioxidant effect on ox-LDL-induced HUVECs.

### Salidroside upregulated expressions of SIRT1 and FOXO1 in HUVECs with ox-LDL

**Fig. 3:**
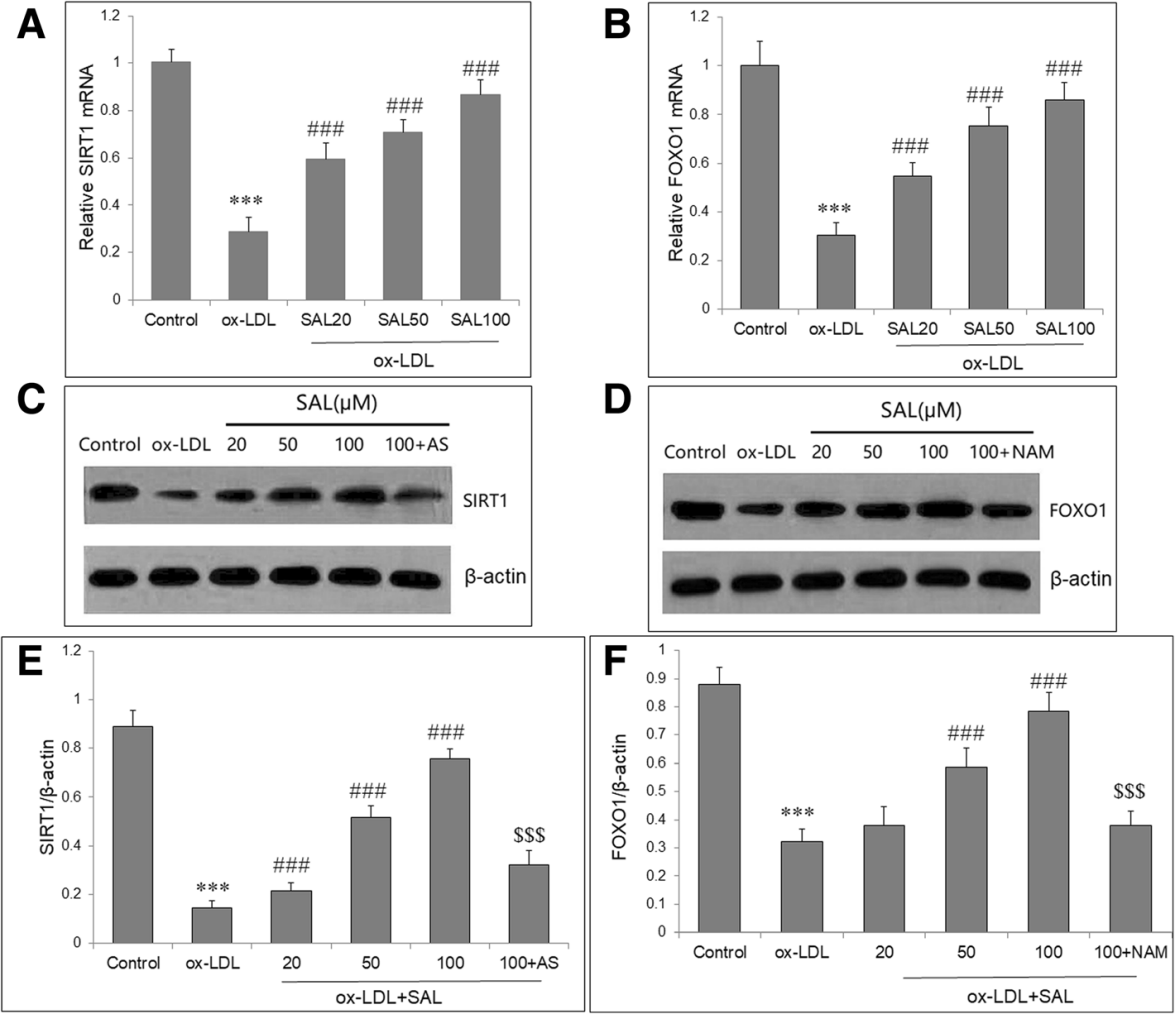
Regulation of SIRT1 and FOXO1 gene by SAL in HUVECs exposed to ox-LDL. HUVECs were pretreated with SAL (20, 50, 100 μM) for 2 h, and then incubated with ox-LDL (100 μg/mL) for 48 h. qRT-PCR shows that the mRNA levels of SIRT1 (**a**) and FOXO1 (**b**) were significantly reduced by ox-LDL, but was increased by SAL in a concentration dependent manner. Western blotting was performed to determine protein levels of SIRT1 (**c**) and FOXO1 (**d**). In HUVECs with SAL (100 μM), cells were pre-incubated with FOXO1 inhibitor (AS1842856, 100 nM), or SIRT1 inhibitor nicotinamide (NAM, 200 nM). SAL significantly attenuates the decrease in SIRT1 (**e**) and FOXO1 (**f**) proteins in HUVECs exposed to ox-LDL. Data are shown as mean ± SD (*n* = 6). ***P < 0.001 vs. control group; ##*P* < 0.01, ###*P* < 0.001 vs. ox-LDL group; $$$*P* < 0.001 vs. SAL (100 μM) group

In order to explore underly mechanisms regulated by SAL, we measured mRNA and protein expressions of two genes which are associated with autophagy, SIRT1 and FOXO1. HUVECs were pretreated with SAL at 20, 50, 100 μM for 2 h and exposed to ox-LDL (100 μg/mL) for further 48 h. qRT-PCR showed that compared with control cells, the mRNA expressions of SIRT1 and FOXO1 were significantly decreased after exposure to ox-LDL (100 μg/mL). Co-treatment with SAL significantly increased SIRT1 and FOXO1 mRNA expressions in a concentration-dependent manner (Fig. 3a, b). Western blotting also showed that SIRT1 and FOXO1 proteins were obviously decreased after exposure to ox-LDL, and were enhanced by SAL pretreatment (Fig. 3c, d). Furthermore, to investigated the mutual regulation between SIRT1 and FOXO1, HUVECs were treated with SAL (100 μM), or with SAL (100 μM) plus FOXO1 inhibitor (AS1842856, 100 nM), or with SAL (100 μM) plus SIRT1 inhibitor nicotinamide (NAM, 200 nM). The results showed that increase in SIRT1 protein by SAL could be attenuated by AS1842856, and increase in FOXO1 protein by SAL could be obviously abolished by NAM (Fig. 3e, f). This indicates that there is inter-regulation between SIRT1 and FOXO1 in SAL-induced endothelial protection and autophagy promotion.

### SIRT1 and FOXO1 mediate endothelial injury, oxidative stress and autophagy by salidroside

**Fig. 4:**
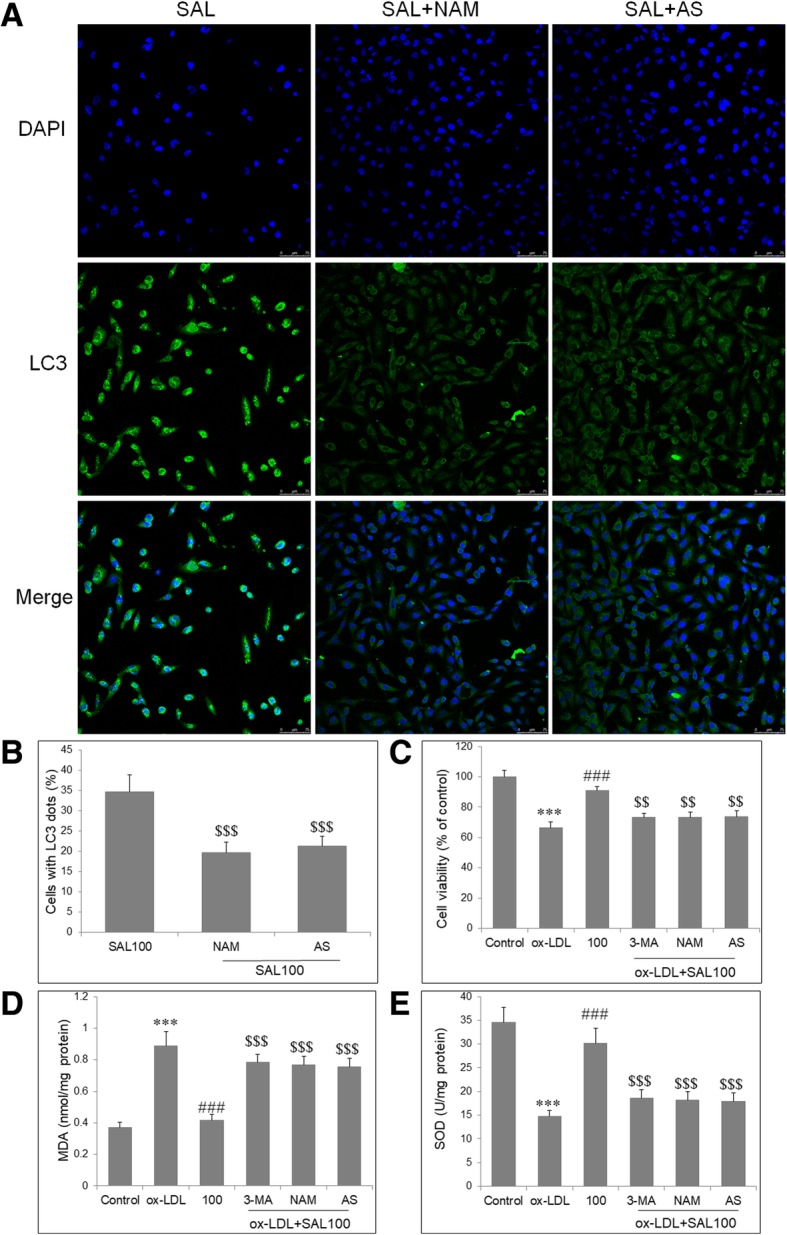
SIRT1 and FOXO1 mediate the protective effects of SAL on ox-LDL-induced endothelial injury, oxidative stress and autophagy. **a** Autophagy of HUVECs stained with an LC3 antibody under fluorescence microscopy. In cells with ox-LDL and SAL (100 μM), pretreatment with NAM (200 nM) or AS1842856 (100 nM) obviously reduced fluorescence intensity. **b** Quantification analysis shows that NAM and AS1842856 both significantly reduced the percentages of cells with LC3 dots. Autophagy inhibitor 3-Methyladenine (3-MA; 100 μM), NAM and AS1842856 all obviously abolish the protective effect of SAL on ox-LDL-induced endothelial injury (**c**), increase in MAD level (**d**), and decrease in SOD activity (**e**). Data are shown as mean ± SD (*n* = 6). ***P < 0.001 vs. control group; ###*P* < 0.001 vs. ox-LDL group; $$*P* < 0.01, $$$*P* < 0.001 vs. SAL (100 μM) group

To investigate the role of SIRT1 and FOXO1 in autophagy induction by SAL, HUVECs were incubated with ox-LDL (100 μg/mL) and SAL (100 μM), in combination with NAM (200 nM) or AAS1842856 (100 nM). Compared with HUVECs with SAL alone, cotreatment with NAM (200 nM) or AS1842856 (100 nM) obviously reduced fluorescence intensity and significantly decreased the percentages of cells with LC3 dots (Fig. 4a, b). We then investigated the role of autophagy, SIRT1 and FOXO1 in protection of endothelial injury and oxidative stress by SAL, by cotreatment with autophagy inhibitor 3-Methyladenine (3-MA; 100 μM), NAM and AS1842856. Compared with HUVECs with ox-LDL and SAL, cells cotreated with 3-MA, NAM or AS1842856 significantly reduced cell viability (Fig. 4c), increased in MAD level (Fig. 4d), and decrease in SOD activity (Fig. 4e) in ox-LDL-induced HUVECs.

## Discussion

In this study, we investigated the protective effect of SAL on ox-LDL-induced endothelial injury. ox-LDL (100 μg/mL) exposure reduced cell viability and increased LDH release, which was attenuated by treatment with SAL (20, 50, 100 μM) for 48 h. In HUVECs exposed to ox-LDL, SAL enhanced autophagy and suppressed oxidative stress. SAL attenuated the reduction in mRNA and protein expressions of SIRT1 and FOXO1 by ox-LDL. SIRT1 inhibitor or FOXO1 inhibitor could abolish the suppression on oxidative stress and endothelial injury and enhancement on autophagy by SAL. Therefore, SAL protects HUVECs against ox-LDL-induced injury through upregulation of autophagy mediated by SIRT1-FOXO1 axis.

In our experiment, HUVECs were incubated with 100 μg/mL of ox-LDL for 48 h, and showed reduced viability. This indicates that ox-LDL induces oxidative injury in endothelial cells, as evidenced by increased MDA and decreased SOD. Thus, HUVECs with ROS-derived oxidative injury could simulate endothelial dysfunction in atherosclerosis. Our data demonstrated that SAL exerted cytoprotective effects on endothelial injury, suppressed oxidative stress and enhanced autophagy. The increased cell viability may be related to increased percent of S phase cells and decreased apoptosis by SAL in macrophages [16]. This is the third report about the effect of SAL on ox-LDL-induced cells. The previous reports showed that SAL suppressed foam cell formation and apoptosis in ox-LDL-induced THP1 cells [16], and prevented ox-LDL-treated endothelial cell senescence by increasing percentage of S phase cells [19]. Up to now, there is no report about the direct action of SAL on oxidized LDL. So SAL might interfere with the signal pathways induced by ox-LDL. For example, SAL prevented cytotoxicity in endothelial cell line EVC-304 and primary retinal endothelial cells induced by endogenous and exogenous hydrogen peroxide, respectively [20, 21]. The underlying mechanisms are increased Bcl2/Bax survival signaling pathway and activation of endogenous antioxidant enzymes, which is supported by our results that SAL attenuated cytotoxicity in HUVECs with decreased MDA and increased SOD.

Our study shows that SAL enhanced autophagy while suppressed oxidative stress in HUVECs induced with ox-LDL. The relationship between autophagy and oxidative stress remains controversial in atherosclerosis. Oxidative stress contributes to atherogenesis through oxidation of LDL, which is cytotoxic to vascular cells. While autophagy is most likely a protective mechanism of cell in response to ox-LDL, thus allows atherosclerotic cells to survive [22]. This protective mechanism could be induced in atherosclerosis, as evidenced by activated autophagy in cultured HUVECs by ox-LDL and degradation of ox-LDL by autophagy activation [8]. Our data showed that ox-LDL increased autophagy in HUVECs, and SAL further enhanced autophagy in HUVECs exposed to ox-LDL. Cotreatment of HUVECs with an autophagy inhibitor 3-Methyladenine (3-MA) could abolish increased cell viability by SAL, and this suggests that autophagy induced by SAL is a protective mechanism against atherosclerosis. Our speculation is supported by another report, in which SAL increased autophagy and decreased apoptosis of HUVECs induced by H_2_O_2_ [18].

This study shows that SAL increased mRNA and protein expressions of SIRT1 in HUVECs exposed to ox-LDL, and SIRT1 inhibition reduced autophagy, and reversed suppression on oxidative stress and endothelial injury. SIRT1 exerts protective roles in atherosclerosis through suppression of endothelial oxidative stress and foam cell formation^10^. SIRT1-mediated autophagy could protect HUVECs against Ox-LDL-induced injury by various agents, and our study adds SAL as another protective autophagy stimulator in HUVECs [23, 24]. Moreover, our study shows that SIRT1 has suppressive effect on oxidative stress, and this effect might be related to autophagy induction. The reason lies in the fact that reduced oxidative stress in HUVECs was also abolished by an autophagy inhibitor 3-MA, and one report that SIRT1-induced autophagy mediated suppressed oxidative stress by SAL in status epilepticus [25]. Thus, in ox-LDL exposed HUVECs, SAL activates SIRT1, autophagy, and inhibits oxidative stress, thus protects against endothelial injury.

Our study also shows that FOXO1 mRNA and protein expressions was increased by SAL in HUVECs exposed to ox-LDL, which mediated enhanced autophagy, and suppressed oxidative stress and endothelial injury. FOXO1 regulates various genes involved in response to oxidative stress, and acts as a potent inhibitor of oxidative stress in various diseases with excessive oxidative stress [13]. Moreover, FOXO1 silencing in mouse endothelial cells prevented atherosclerosis, and indicates that FOXO1 is an atheroprotective molecule [14]. FOXO1 could protect cardiomyocytes against oxidative stress-induced apoptosis through inducing autophagy, which is consistent with our results that FOXO1 inhibitor abolished the autophagy induction and attenuated endothelial injury by SAL [26]. Furthermore, SIRT1 and FOXO1 interact with each other in response to oxidative stress. SIRT1 deacetylates FOXO1 and regulates its transcriptional activity, and FOXO1 increases SIRT1 transcription and expression through binding to SIRT1 promoter [27]. This mechanism is also confirmed by our study that in HUVECs with ox-LDL and SAL treatment, increase in SIRT1 protein was attenuated by FOXO1 inhibitor AS1842856, and increase in FOXO1 protein was also attenuated by SIRT1 inhibitor NAM. In fact, activation of SIRT1- FOXO1 signaling axis could inhibit oxidative stress in ischemia/reperfusion injury of heart [28], and enhance autophagy in protecting against apoptosis of osteoblast and reducing cellular senescence in skin exposed to UV irradiation [29, 30]. However, this SIRT1-FOXO1 regulatory loop can be disrupted by oxidative stress, thereby contributing to endothelial dysfunction such as aging and ischemia. The roles and mechanisms of SIRT1-FOXO1 regulatory loop in atherosclerosis remains unknown.

## Conclusion

The present study shows that salidroside enhanced autophagy through upregulation modulation of SIRT1-FOXO1 axis in HUVECs with ox-LDL exposure. The cross talk between autophagy and oxidative stress might contribute to the autophagic protection of HUVECs by salidroside. These findings suggest a novel role for salidroside in inducing the protective autophagy, which may be a potential therapeutic strategy in prevention of atherosclerosis.

## Data Availability

Data and materials are available upon request to the corresponding author.

## Abbreviations

3-MA: 3-Methyladenine
CCK-8: Cell counting kit-8
FOXO1: Forkhead box O1
HUVECs: Human umbilical vein endothelial cells
LDH: Lactate dehydrogenase
LDL: Low density lipoprotein
MDA: Malondialdehyde
NAM: Nicotinamide
ox-LDL: Oxidized low-density lipoprotein
qRT-PCR: Quantitative real-time polymerase chain reaction
SAL: Salidroside
SOD: Superoxide dismutase
